# Astrocytic Calcium Signaling Toolkit (astroCaST): efficient analysis of dynamic astrocytic calcium events

**DOI:** 10.3389/fncel.2024.1408607

**Published:** 2024-06-10

**Authors:** Jan Philipp Reising, Ana Cristina Gonzalez-Sanchez, Athina Samara, Eric Herlenius

**Affiliations:** ^1^Department of Women's and Children's Health, Karolinksa Institutet, Stockholm, Sweden; ^2^Astrid Lindgren Children's Hospital, Karolinska University Hospital, Stockholm, Sweden; ^3^Department of Biomaterials, FUTURE, Center for Functional Tissue Reconstruction, University of Oslo, Oslo, Norway

**Keywords:** astrocytes, toolkit, calcium, timeseries, detection, events

## Abstract

The Astrocytic Calcium Signaling Toolkit (astroCaST) is a novel solution to a longstanding challenge in neuroscience research: the specialized analysis of astrocytic calcium events within fluorescence time-series imaging. Distinct from existing neuron-centric tools, astroCaST is adept at detecting and clustering astrocytic calcium events based on their unique spatiotemporal characteristics, thus filling a gap in astrocytic research methodologies. This toolkit not only facilitates the detection of such events but also extends its utility to provide comprehensive end-to-end analysis. This feature is absent in most tools targeting astrocytic activity. AstroCaST's development was motivated by the critical need for dedicated software that supports researchers in transitioning from raw video data to insightful experimental conclusions, efficiently managing large-scale datasets without compromising computational speed. It offers a user-friendly interface that caters to both novice and expert users, incorporating both a graphical user interface (GUI) for detailed explorations and a command-line interface (CLI) for extensive analyses. Expected outcomes from utilizing astroCaST include the ability to process and analyze a significantly larger volume of data. This enables a more profound and comprehensive analysis than previously possible, addressing the demands of large-scale astrocytic studies. In summary, astroCaST aims to advance astrocytic calcium imaging analysis, offering a tailored, efficient, and comprehensive toolset that enhances our understanding of astrocytic functions and their implications in neuroscience.

## 1 Introduction

Analyzing cytosolic calcium oscillations helps decode neuronal firing patterns, synaptic activity, and network dynamics, offering insights into cell activity and states (Del Negro, 2005; Dombeck et al., 2007; Grienberger and Konnerth, 2012; Forsberg et al., 2017). Furthermore, the changes in calcium activity may be indicative of cell responses to downregulation of molecular pathways, epigenetic alterations, or even the effect of treatments or drugs, and disease states (Zhang et al., 2010; Huang et al., 2013; Robil et al., 2015; Britti et al., 2020; Lines et al., 2020; Miller et al., 2023). This makes dynamic calcium activity recordings a crucial tool to investigate physiology and neurological disorders and to design and develop therapeutic interventions.

While we have seen major development in recent years in the imaging tools available to study astrocytes, the software side has been slow to catch up (Stobart et al., 2018a; Aryal et al., 2022; Gorzo and Gordon, 2022). Currently several software packages are available for researchers to analyze calcium activity recordings from brain cells (Table 1). However, most of these packages are primarily developed for neurons, and are often not suitable for astrocytes. The challenge stems from astrocytes' unique physiology, marked by rapid microdomain calcium fluctuations (Stobart et al., 2018b; Curreli et al., 2022) and their ability to alter the morphology during a single recording (Baorto et al., 1992; Anders et al., 2024). Astrocytes exhibit calcium fluctuations that are spatially and temporally diverse, reflecting their integration of a wide range of physiological signals (Smedler and Uhlén, 2014; Bazargani and Attwell, 2016; Papouin et al., 2017; Denizot et al., 2019; Semyanov et al., 2020).

**Table 1:** Comparison of computational tools for analyzing cellular calcium oscillations.

	**Cell type**	**Model**	**Validation**	**Imaging**	**Language**	**Modular**	**GUI**
Suite2P (Pachitariu et al., 2017)^*^	Neurons	*In-vivo*	Comparison	2P	Python	✓	✓
FASP (Wang et al., 2017)	Astrocytes	*In-vitro*	Synthetic	—	Java	✓	✓
CHIPS (Barrett et al., 2018)	Endothelial	*In-vitro*	—	2P confocal	Matlab	✓	✓
		*In-vivo*					
AQuA (Wang et al., 2018)	Astrocytes	*In-vitro*	Synthetic	2P	Matlab	—	✓
		*In-vivo*					
CaImAn (Giovannucci et al., 2019)	Neurons	*In-vitro*	User labels	1P, 2P	Python	✓	✓
		*In-vivo*					
Astral (Dzyubenko et al., 2021)	Astrocytes	*In-vitro*	—	1P	Python	—	✓
Begonia (Bjørnstad et al., 2021)	Astrocyte	*In-vivo*	—	2P	Matlab	✓	✓
CaSCaDe (Rupprecht et al., 2021)	Neurons	*In-vivo*	Ground truth	1P, 2P	Python	✓	—
		*In-vitro*					
		*Ex-vivo*					
MSparkles (Stopper et al., 2022)^*^	Astrocytes	*In-vivo*	Comparison	1P, 2P	Matlab	—	✓
	Neurons	*Ex-vivo*					
astroCaST	Astrocytes	*In-vivo*	Synthetic	1P, 2P	Python	✓	✓
		*Ex-vivo*					

This table highlights the diversity of approaches, including the programming language used, modularity of the tool, and the availability of a graphical user interface (GUI). Modular indicates whether steps can be used in isolation and custom steps can be added. Notably, entries marked with an asterisk (*) are based on preprint sources.

The available toolkits specifically transcribing astrocytic activity come with several shortcomings (Table 1). A major barrier is the use of proprietary programming languages like Matlab, that hinder widespread use. Additionally, slow implementations due to lack of parallelization and Graphics Processing Unit (GPU) acceleration or usage of RAM exceeding most standard setups impedes analysis of long videos (>5,000 frames). While all compared toolkits offer efficient event detection, most lack support to gain insight from the extracted events.

AstroCAST, developed in Python for its versatility and ease of use, addresses these shortcomings through a modular design, allowing for customizable pipelines and parallel processing. It optimizes resource use by only loading data as needed, ensuring efficient hardware scaling. Its modular design enables stepwise quality control and flexible customization. Finally, astroCaST includes dedicated modules to analyze common research questions, as compared to other packages that primarily focus on event detection.

**Figure 1:**
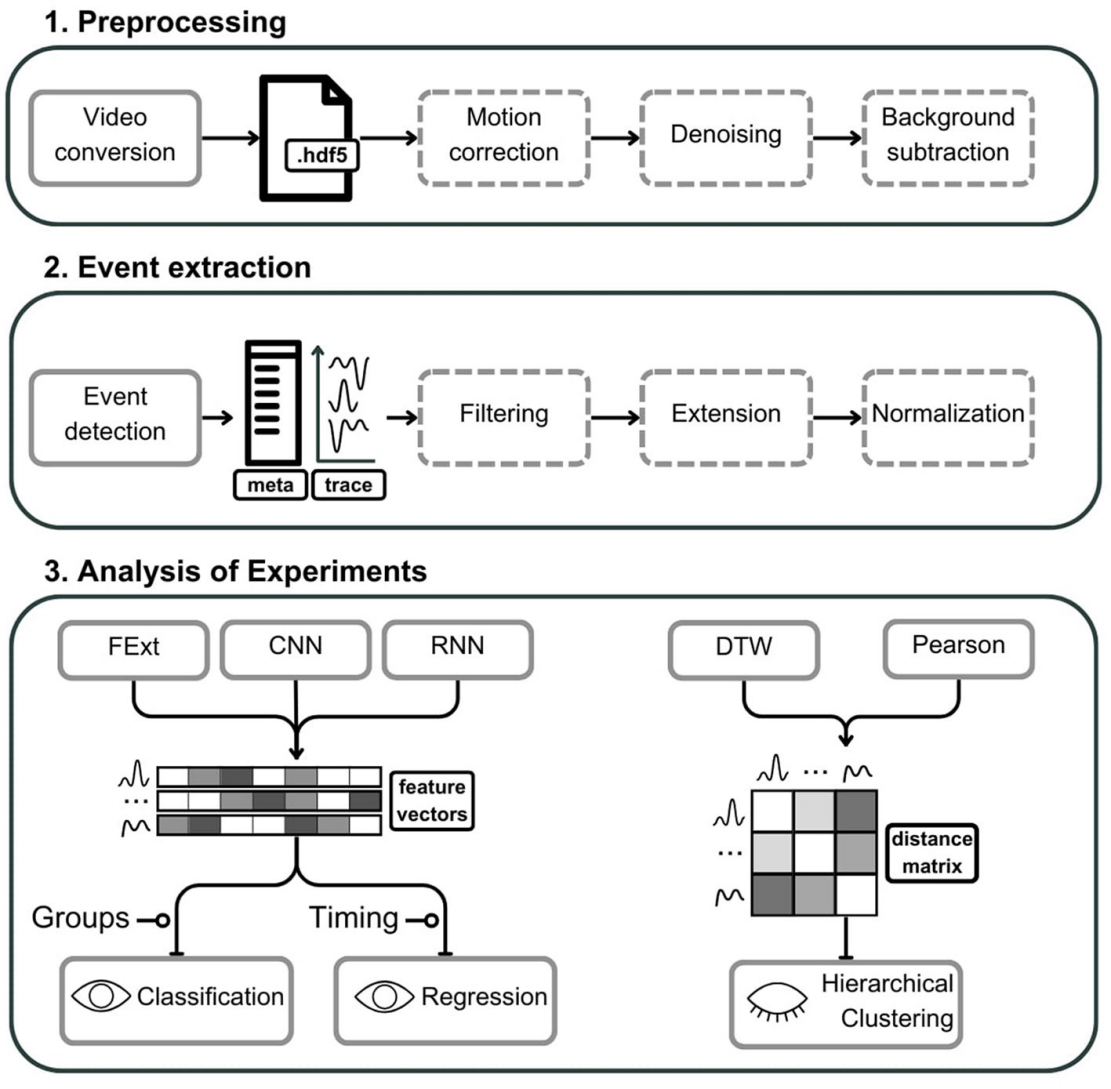
Schematic representation of the astroCaST toolkit comprising three major analytical phases for processing and analyzing astrocytic activity data. (1) In the preprocessing phase, video input is converted into an HDF5 file format, with optional stages for motion artifact correction, noise reduction, and background subtraction. (2) The event extraction phase involves the identification of astrocytic events, generating corresponding event traces and metadata. Events can subsequently undergo optional filtering, frame extension, and signal normalization. (3) The analysis of experiments is two-fold. For classification and regression, users may select from various feature vector embeddings including Feature Extraction (FExt), Convolutional Neural Network (CNN), and Recurrent Neural Network (RNN). For hierarchical clustering, the toolkit offers Pearson correlation and Dynamic Time Warping (DTW) for the computation of event similarity, represented as a distance matrix. Dashed outlines denote optional steps within the process. The presence of an open eye icon signifies supervised steps, while a closed eye indicates unsupervised steps. A line terminating in a circle denotes need for additional user input.

Here, we not only introduce astroCaST but also provide a detailed guide for extracting astrocytic calcium signals from video data, performing advanced clustering and correlating their activity with other physiological signals (Figure 1). AstroCAST extends beyond theory, having been tested with synthetic data, as well as *in-vitro, ex-vivo*, and *in-vivo* recordings (Table 3). This underscores AstroCAST's ability to harness sophisticated computational techniques, making significant strides in the study of astrocytes and offering new opportunities for neuroscience research.

## 2 Methods

### 2.1 Requirements

It is imperative that the recordings specifically capture astrocytes labeled with calcium sensors, either through transgenic models or viral vectors. While the use of calcium dyes is possible, their application may not guarantee the exclusive detection of astrocytic events. The initial step of the astroCaST toolkit involves preparing the video recordings for analysis. Our protocol supports a range of file formats, including .avi, .h5, .tiff (either single or multipage), and .czi, accommodating videos with interleaved recording paradigms. To ensure reliable results, recordings should be captured at a frequency of at least 8 Hz for One-photon (1P) imaging and 1–2 Hz for Two-photon (2P) imaging. This frequency selection is crucial for capturing events of the expected duration effectively.

Regarding hardware requirements, our protocol is adaptable to a variety of configurations, from personal computers to cloud infrastructures, with certain modules benefiting from GPU acceleration. At a minimum, we recommend using hardware equipped with at least 1.6 GHz quad-core processor and 16 GB of RAM to efficiently handle the data analysis. In cases where the available memory is a bottleneck, astroCaST offers a lazy parameter to only load relevant sections of the data into memory. While this increases processing time, depending on the speed of the storage medium, it allows users to analyze datasets that would usually exceed the capabilities of their hardware.

On the software side, we advocate for the use of the Linux operating system, specifically Ubuntu or AlmaLinux distributions, for optimal performance. However, Windows or macOS can be used with some functional limitations (Table 2). Be advised, that the M1 and M2 Mac processors are currently not supported. Essential software includes Python version 3.9, Anaconda or Miniconda, and git for version control. Furthermore, we recommend the use of ImageJ or an equivalent image viewer for analyzing the output visually. This comprehensive approach ensures that researchers can accurately extract and analyze astrocytic calcium signals, paving the way for further understanding of their physiological roles.

**Table 2:** Availability of different functionalities of astroCaST across operating systems.

**Functionality**	**Linux**	**Windows**	**MacOS (Intel)**	**Docker**
Preprocessing	✓	✓	✓	✓
Motion correction	✓	✓	✓	✓
Denoising	✓	◐	✓	✓
Delta	✓	✓	✓	✓
Detection	✓	✓	✓	✓
UMAP outlier detection	✓	✓	—	✓
Encoding - all	✓	✓	✓	✓
DTW distance	✓	✓	—	✓
Video player *	✓	✓	✓	—

A check mark (✓) denotes full compatibility and active support. An asterisk (*) indicates that the functionality is optional and can be installed upon user request (see below). A cross mark (—) signifies that the functionality is not supported or available on the operating system. A half bullet (◐) indicates partial support, indicating that the functionality is expected to be available but is not actively tested.

### 2.2 Installation

To run the software, astroCaST and its dependencies must be installed. There are multiple options to do this depending on how much control users would like to have over the installation. Of note, the following instructions install astroCaST with its full functionality. If that is not desired or possible remove the -E all or [all] flags.

#### 2.2.1 Creating a conda environment (optional)

While not strictly necessary, we highly recommend to create a fresh anaconda environment. This prevents common installation errors and conflicts with existing environments.


          > conda create -n astro python=3.9 poetry
          > conda activate astro

#### 2.2.2 Install from source (recommended)


         > **cd** ''/path/to/directory/''
         >   git clone git@github.com:janreising/astroCAST.git
         > **cd** astroCAST
         > poetry   install -E all

#### 2.2.3 Installation with pip (easiest)


          # *install core features*
          > pip install astrocast
          # *install with all features*
          > pip install astrocast[all]

#### 2.2.4 Container installation (last resort)

To install docker and create an account, follow the instructions on the docker webpage: https://docs.docker.com/engine/install/


          > docker pull anacgon/astrocast:latest
          > docker image ls
          > docker run -v /path/to/your/data:/home/data -it
          -p 8888:8888 astrocast:latest
          # *Optionally, start jupyter notebook from inside*
          the docker container:
          > jupyter-lab --allow-root --no-browser
          --port=8888 --ip=''*''

#### 2.2.5 Test installation

Both commands should run without any errors.


          > python -c ''import⌴astrocast''
          > astrocast --help

### 2.3 Animal models

Our research utilized two transgenice mouse strains whose background was changed to outbred CD-1 mice supplied by Charles River Laboratories, located in Germany. The initial transgenic lines were Aldh1l1-Cre (JAX Stock No. 029655: B6N.FVB-Tg(Aldh1l1-cre/ERT2)1Khakh/J) and Ai148D mice (Jax Stock No. 030328: B6.Cg-Igs7tm148.1(tetO-GCaMP6f,CAG-tTA2)Hze/J). These strains were housed in a controlled environment featuring a cyclical light-dark period lasting 12 hours each. Unlimited access to food and water was ensured. These mice were housed at the Department of Comparative Medicine at Karolinska Institutet in Stockholm, Sweden. Our experimental protocols were in strict compliance with the European Community Guidelines and received authorization from the Stockholm Animal Research Ethics Committee (approval no. 15819-2017). Acute slices from the pre-Bötzinger to the protocol previously established by Reising et al. (2022).

### 2.4 Datasets used during development

AstroCAST was developed using publicly available datasets and our own data. The data represents astrocytes studied *ex-vivo, in-vivo* and in acute slices captured with 1P or 2P (Table 3). To ensure transparency and provide a practical starting point, we offer a collection of tested, default settings within a YAML file. This file, designed to represent a first-approach configuration, is accessible in our GitHub repository. Additionally, we provide the datasets used in this manuscript, as well as pretrained models. A curated list of pretrained models is accessible on our GitHub page under denoiser models, and a comprehensive collection of models can be downloaded via astroCaST. The file name of the model summarizes the model and training parameters.

*          # download public and custom datasets*
          > astrocast download-datasets
          ''/path/to/download/directory''
  
*          # download pretrained models*
          > astrocast download-models
          ''/path/to/download/directory''

**Table 3:** Overview of datasets employed in analysis.

**Dataset**	**Publication**	**Experiment**	**Imaging**
Train/test	astroCaST (section 2.4)	Acute slices, GCaMP6f	1P (8 Hz)
*ExVivo*	AQuA (Wang et al., 2018)	Acute slices, GCaMP6f	2P (1.1 Hz)
Glusnfr	AQuA (Wang et al., 2018)	Acute slices, GluSnFR	2P (4–100 Hz)
*InVivo*	AQuA (Wang et al., 2018)	*In vivo* GCaMP6f	2P (2 Hz)
Cellscan_scim	CHIPS (Barrett et al., 2018)	Blood vessels	2P

### 2.5 Benchmarking of astroCaST and AQuA

A novel synthetic video dataset was designed to evaluate the performance of both astroCaST and AQuA. This dataset was specifically developed following the implementation of astroCaST, and was not used for the optimization of astroCaST. The choice of a synthetic dataset is justified due to the lack of suitable publicly accessible datasets with verified ground truth labels. Both algorithms were run with their default settings, astroCaST with settings from config.yaml and AQuA with preset 2 (*ex-vivo*), to ensure unbiased performance comparison. The computational analysis was conducted on the Rackham HPC cluster at UPPMAX, Uppsala University, employing 12 CPU cores (Intel Xeon V4), 76.8 GB of RAM, and 20 hours of allocated wall time. A detection was considered failed if it surpassed either the memory capacity or time constraints. Within these parameters, AQuA successfully processed video dimensions up to 5,000 × 800 × 800 pixels, whereas astroCaST managed videos up to 5,000 × 1,200 × 1,200 pixels. Events were filtered to lengths between 5 and 1,000 frames to eliminate evident anomalies.

## 3 Results

### 3.1 Using astroCaST

AstroCAST is designed to guide users through the entire process of astrocyte calcium analysis, from the initial raw data to in-depth analysis. It is a versatile toolkit that allows for a customizable workflow as users have the flexibility to omit certain steps or integrate additional analyses as their research demands. AstroCAST serves as a robust companion for computational exploration in neuroscience.

Central to the toolkit's utility is the astroCaST Python package, which offers direct access to all functionalities of the toolkit. This enables users to incorporate astroCaST seamlessly into their existing workflows or to automate processes within their custom scripts. The toolkit is structured into three major blocks: Preprocessing, Encoding, and Exploratory Analysis.

In the Preprocessing block, users can use a CLI, accessible via commands such as astrocast--help directly in the terminal. The CLI facilitates the use of a predefined configuration file to streamline the preprocessing tasks. Moreover, an intuitive “Argument Explorer” is incorporated to assist users in quickly testing different parameters, enabling export of the resultant configuration settings. For a hands-on introduction to this stage, we have provided a Jupyter notebook, allowing users to engage with the protocol interactively.

The Encoding and Analysis block adapts to the specific needs of individual experiments, catering to diverse research objectives such as comparing drug treatments, model systems, correlating activities with stimuli, or monitoring changes over time. This phase of the pipeline is supported by a dedicated GUI, which offers an interactive environment for analysis and enhanced data visualization capabilities.

Acknowledging the diversity in data analysis approaches within astroCaST, subsequent sections will provide concise guidance to interact with astroCaST and ensure that users can harness its full potential.

#### 3.1.1 Jupyter notebooks

Jupyter notebooks can be used to interactively run the analysis and visualize the results. If you have cloned the repository (Section 2.2.2), an example notebook is included to follow along with the steps described here.


          > **cd** /path/to/astroCAST/notebooks/examples/
          > jupyter lab
*          # on MacOS the command might be*
          > jupyter-notebook

A browser window will open which displays the available examples or can be used to create custom notebooks.

#### 3.1.2 The command line interface (CLI)

The CLI is a useful selection of commands that enables users to perform common analysis steps directly from the terminal. Especially in the context of high-performance computing this way of interacting with astroCaST is convenient. All parameters can either be provided manually in the terminal or through a configuration YAML file. We recommend using a configuration file when preprocessing many videos to ensure that the results can be compared. The configuration file must adhere to the YAML format and can contain settings for more than one command. A default configuration file can be found in the GitHub repository.

*          # get list of all available commands*
          > astrocast --help
*          # get help for individual commands*
          > astrocast COMMAND --help
*          # use manual settings*
          > astrocast COMMAND --PARAM-1 VALUE1 [...]
*          # use a configuration file*
          > astrocast --config ‘/path/to/config' COMMAND
          [...]

#### 3.1.3 The graphical user interface (GUI)

AstroCAST implements two GUIs, based on shiny (Chang et al., 2024), to simplify the selection of suitable parameters for the analysis. The commands below will either automatically open a browser page to the interface or provide a link that can be copied to a browser.


         # Identify correct settings for the event detection
         > astrocast explorer -- input path /path/to/file
         -- h5-loc /dataset/name
         # Explore detected events, including filtering,
         embedding and experiments
         > astrocast exploratory-analysis -- input path
         /path/to/roi/ -- h5-loc /dataset/name

#### 3.1.4 The data viewer

For convenience astroCaST includes a data viewer, based on napari (Ahlers et al., 2022), which allows for fast and memory efficient visualization of data. Users can default to their image viewer of choice, like ImageJ, if they choose to do so.

*          # view single dataset*
          > astrocast view-data --lazy False /path/to/file
          /dataset/name
*          # view multiple datasets*
          > astrocast view-data /path/to/file /dataset/one
          /dataset/two
*          # view results of detection*
          > astrocast view-detection-results --video-path
          /path/to/video --loc /dataset/name /path/to/dir/
          name.roi

### 3.2 Preprocessing

#### 3.2.1 File conversion

AstroCAST is designed to handle a wide range of common input formats, such as .tiff and .czi files, accommodating the diverse nature of imaging data in neuroscience research. One of the key features of astroCaST is its ability to process interleaved datasets. By specifying the –channels option, users can automatically split datasets based on imaging channels, which is particularly useful for experiments involving multiple channels interleaved, such as alternating wavelengths.

Moreover, astroCaST supports the subtraction of a static background from the video recordings using the –subtract-background option. This feature allows for the provision of a background image or value, which is then subtracted from the entire video. Subtracting for example the dark noise of the camera can significantly enhance the quality of the analysis.

For optimal processing and data management, it is recommended to convert files to the .h5 file format. The .h5 format benefits from smart chunking, enhancing the efficiency of data retrieval and storage. The output configuration can be finely tuned with options such as –h5-loc for specifying the dataset location within the .h5 file, –compression for selecting a compression algorithm (e.g., “gzip”), and –dtype for adjusting the data type if the input differs from the intended storage format.

Chunking is a critical aspect of data management in astroCaST, allowing for the video to be divided into discrete segments for individual saving and compression. The –chunks option enables users to define the chunk size, balancing between retrieval speed and storage efficiency. An appropriately sized chunk can significantly improve processing speed without compromising on efficiency. In cases where the optimal chunk size is uncertain, setting –chunks to None instructs astroCaST to automatically determine a suitable chunk size.

Lastly, the –output-path option directs astroCaST where to save the processed output. While the .h5 format is recommended for its efficiency, astroCaST also supports saving in formats such as .tiff, .tdb, and .avi, providing flexibility to accommodate various research needs and downstream analysis requirements.

*          # (optional) browse the available flags*
          > astrocast convert-input --help
*          # ‘--config' flag can be ommited to use default*
          settings
          > astrocast --config ''/path/to/config''
          convert-input ''/path/to/file/or/folder''

**Figure 2:**
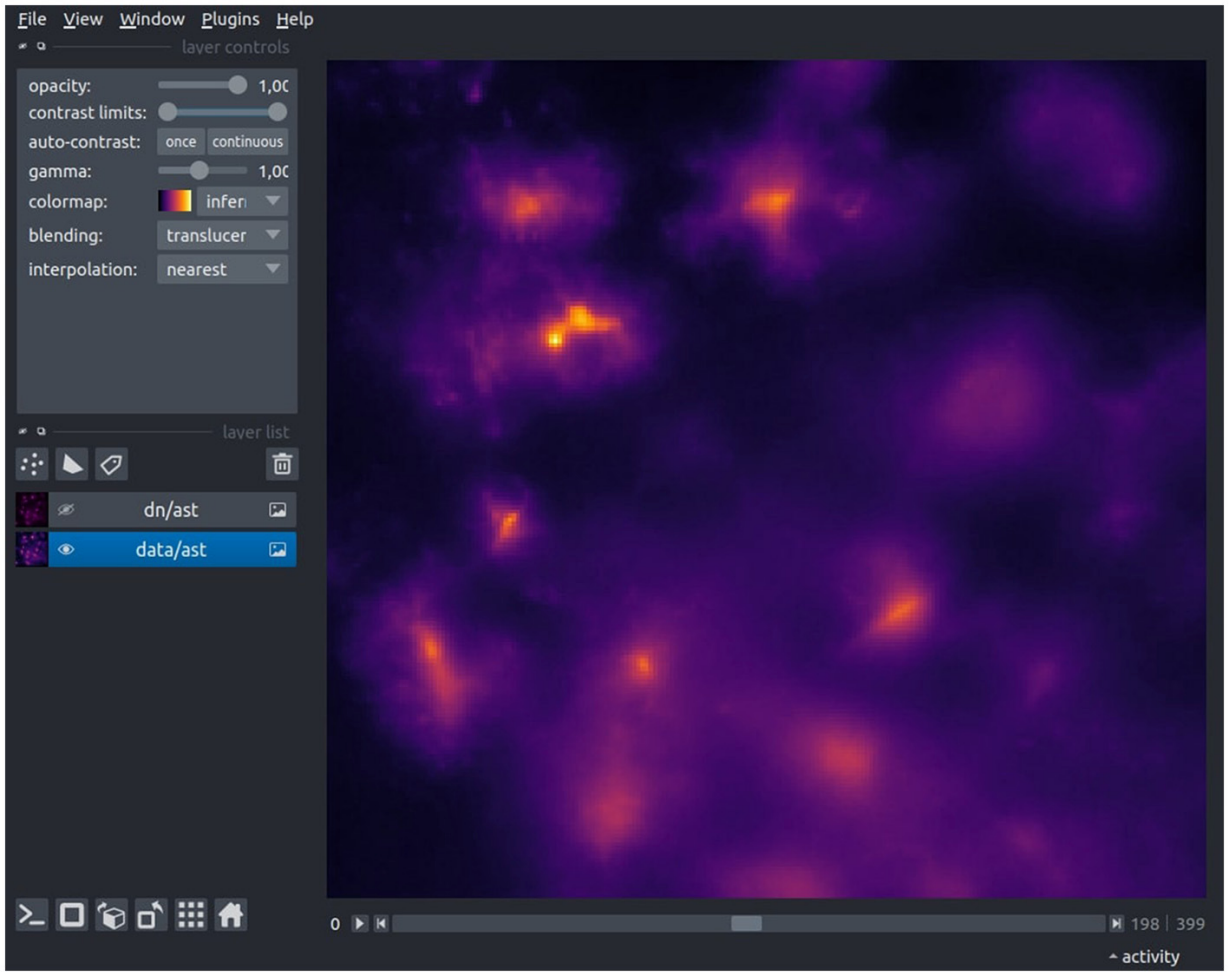
The Astrocast Viewer interface, showcasing a video file of astrocytic calcium fluorescence captured within the inspiratory rhythm generator (preBötC) in the medulla. The original recording, acquired at a 20X magnification with a resolution of 1,200 × 1,200 pixels, was performed at a temporal resolution of 8 Hz. The TIFF-format video was subsequently converted to the HDF5 file format, with spatial downscaling by a factor of four in the XY plane, as a preparatory step for further preprocessing.


We recommend to verify the conversion through a quick visual inspection using the built-in data viewer (Figure 2) or an imaging software of your choice (e.g., ImageJ). During this check, ensure that the pixel values are within the expected range (int or float), the image dimensions (width and height) are as anticipated, all frames have been successfully loaded, de-interleaving (if applicable) has been executed correctly, and the dataset name is accurate.

          > astrocast view-data --h5-loc ''dataset/name''
          ''/path/to/your/output/file''

#### 3.2.2 Motion correction (optional)

During imaging, samples will drift or warp which means that the location of the astrocytes might change in the Field of View (FOV). Depending on the amount of movement, this can have detrimental consequences to the analysis. The motion correction module of the CaImAn package (Giovannucci et al., 2019), is used in the astroCaST protocol to correct these artifacts. We refer users to the the jnormcorre documentation for detailed information, but explain the commonly used parameters here.

A cornerstone of this feature is the ability to set the maximum allowed rigid shift through the –max-shifts parameter. This parameter is critical for accommodating sample motion, ensuring that shifts do not exceed half of the image dimensions, thus balancing between correction effectiveness and computational efficiency. The precision of motion correction is further refined using the –max-deviation-rigid option, which limits the deviation of each image patch from the frame's overall rigid shift. This ensures uniformity across the corrected image, enhancing the accuracy of the motion correction process. For iterative refinement of the motion correction, astroCaST employs the –niter-rig parameter, allowing up to three iterations by default. This iterative approach enables a more accurate adjustment to the motion correction algorithm, improving the quality of the processed images. To address non-uniform motion across the field of view, astroCaST offers the piecewise-rigid motion correction option, activated by setting –pw-rigid to True. This method provides a more nuanced correction by considering different motion patterns within different image segments, leading to superior correction outcomes. Furthermore, the –gsig-filt parameter anticipates the half-size of cells in pixels. This information aids in the filtering process, crucial for identifying and correcting motion artifacts accurately.

By carefully adjusting these parameters, researchers can significantly enhance the quality of their imaging data, ensuring that motion artifacts are minimized and that the resultant data is of the highest possible accuracy for subsequent analysis.

*          # ‘--config' flag can be ommited to use default*
          settings
          > astrocast --config ''/path/to/config'' motion-
          correction --h5-loc ''dataset/name''
          ''/path/to/file''

#### 3.2.3 Denoising (optional)

Denoising is an optional but often beneficial preprocessing step in image analysis. The denoising module is based on the architecture suggested for DeepInterpolation (Lecoq et al., 2021) and employs a Convolutional Neural Network (CNN) designed to clean a target frame. The CNN uses adjacent frames as a reference without exposing it to the target frame itself. This approach enables the network to interpolate the desired signal from the contextual frames. Since the noise is stochastic, the network inherently learns to disregard it, effectively isolating and enhancing the signal in the process. We have extended the original approach to support videos of varying dimensions and facilitate straightforward retraining protocols.

Training the denoising model is typically a singular task; once the model is trained, it can be applied to multiple datasets with no further adjustments. The duration of this initial training varies from 1 to 12 h, contingent on data intricacy and available computational power. Subsequent application of the model to new data, known as inference, is considerably more expedient, usually requiring minutes for each file. For convenience, we offer a suite of pre-trained models tailored to different imaging modalities (Section 2.4).

##### 3.2.3.1 Training (optional)

To ensure the robustness and reliability of the denoising process, it is crucial to carefully select the training dataset for the denoiser. The approach to training should aim to minimize the model's exposure to the data it will denoise, which can enhance the reliability of denoising and prevent overfitting.

One approach is to train the model on a separate data set that will not be used in subsequent analysis. This method is advantageous because it allows the model to learn from a diverse set of data, ideally encompassing all experimental conditions expected in the study. However, it requires having a separate dataset that is representative of the various modalities, which may not always be available.

For smaller datasets or when data is limited, a transformation-based approach can be applied. Training on all experimental data but using rotated frames (90°, 180°, and 270°) ensures that the denoiser is exposed to the inherent noise and variability in the data while mitigating direct exposure to the frames being denoised. This approach is particularly resource-efficient but may result in a less robust model due to the potential predictability of rotated frames.

Each of these methods has its merits and should be chosen based on the specific context and availability of data within a given study.

*          # explore the possible parameters*
          > astrocast train-denoiser --help
*          # train model from config file*
          > astrocast --config config.yaml train-denoiser

The intricacies of training a custom model are beyond this protocol, and we refer to the original DeepInterpolation publication (Lecoq et al., 2021) and our example notebook for users interested in training their own model.

##### 3.2.3.2 Denoising data

**Figure 3:**
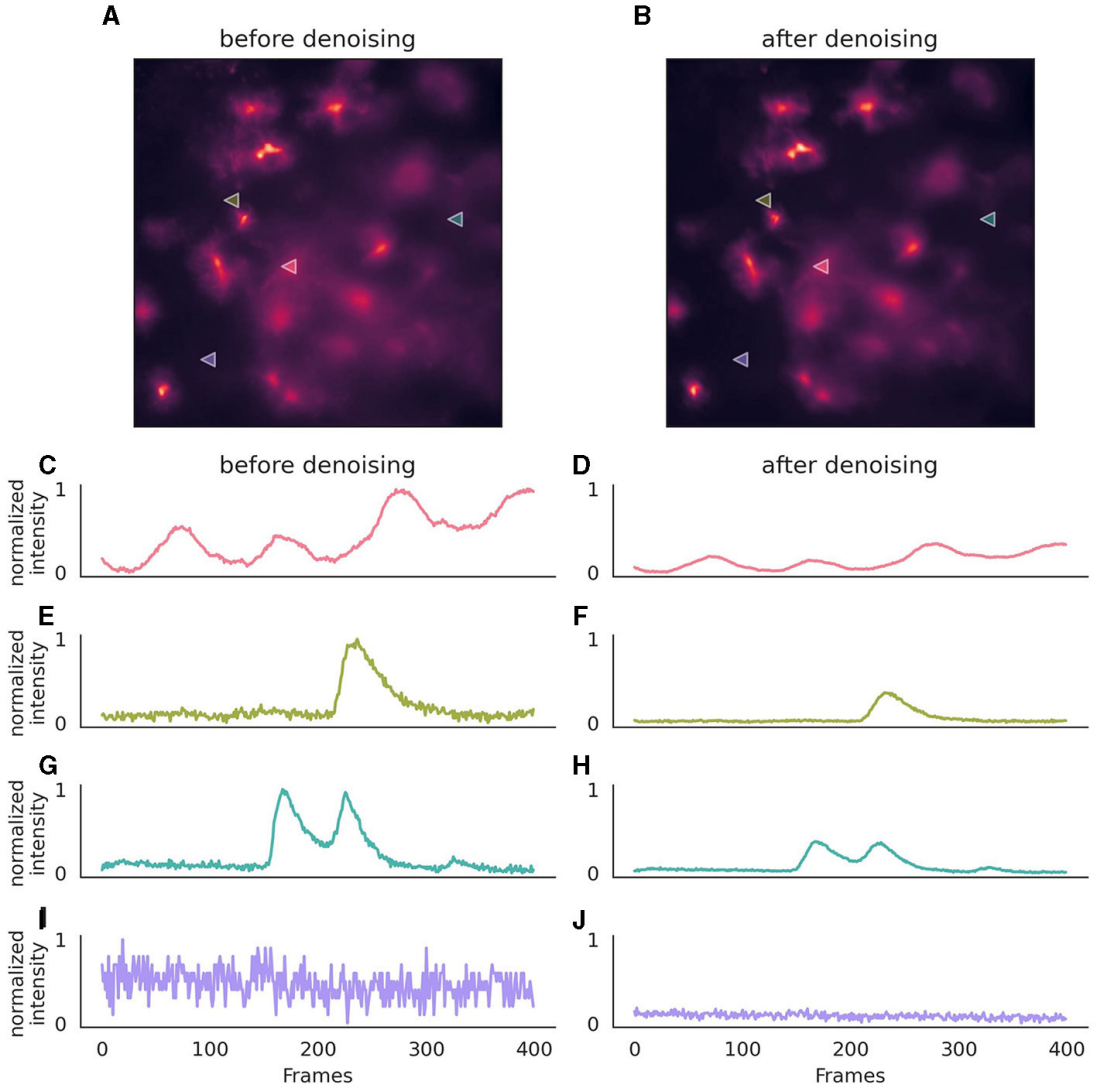
Effect of denoising on astrocytic calcium imaging data. **(A)** A single 256 × 256 pixel frame prior to denoising, where background noise is evident. **(B)** The same frame following denoising with enhanced clarity. **(C, E, G, I)** Pixel intensity over 400 frames (50s) before the application of the denoising algorithm, showcasing the original signal variation, after normalization. **(D, F, H, J)** Corresponding traces after denoising, illustrating a stabilized intensity profile, with preserved signal characteristics. The same normalization parameters were applied to both original and denoised traces to ensure comparability of noise levels. Location of pixels are indicated with triangles in **(A, B)**. The denoising algorithm was executed on a (128, 128) field of view, incorporating a context of five adjacent frames for each target frame, with no gap frames. Parameters for the denoising model included a training period of 50 epochs, a learning rate of 0.0001, momentum of 0.9, and a stack of three layers with 64 kernels of size three in the initial layer, omitting batch normalization. During inference, a strategy of a 10-pixel overlap in all directions, complemented by “edge” padding, was employed.

Once the neural network has been trained for denoising, it can be employed on new data using the parameters that were set during its training (Figure 3). If utilizing a provided pre-trained model, these parameters are inferred from the model file.

For trivial input parameters, such as the model path and output file location, the following flags are used: –model to specify the model, –output-file to define the output file, –loc-in and –loc-out to designate the input and output data locations within the HDF5 file structure.

Network architecture parameters are crucial for ensuring that the denoising process is compatible with the data's specific attributes. These parameters need to exactly match the parameters during training. These include –input-size to set the dimensions of the input data, –pre-post-frames to determine the number of frames before and after the target frame used during denoising, –gap-frames to optionally skip frames close to the target frame, and –normalize to apply normalization techniques that aid the network in emphasizing important features over noise.

Image parameters cater to the post-processing needs of the denoised data. The –rescale parameter is used to reverse the normalization applied during denoising, as subsequent analysis steps often expect the pixel values to be in their original scale. Furthermore, –padding adjusts the input data size to match the network's expected input dimensions, ensuring that no data is lost or distorted during the denoising process.

While denoising imaging data can reduce noise, it is optional and careful consideration of research needs beforehand ensures that important biological signals aren't inadvertently removed. It may not suit analyses of fast transient signals (<300 ms) as it might obscure crucial biological events (Héja et al., 2021; Cho et al., 2022; Georgiou et al., 2022). Notably, increasing acquisition speed can help preserve rapid events, as it provides the denoising model with more frames to accurately identify rapid changes.

*          # load a config file with parameters*
          > astrocast --config config.yaml denoise
          ''/path/to/file''

#### 3.2.4 Background subtraction (optional)

**Figure 4:**
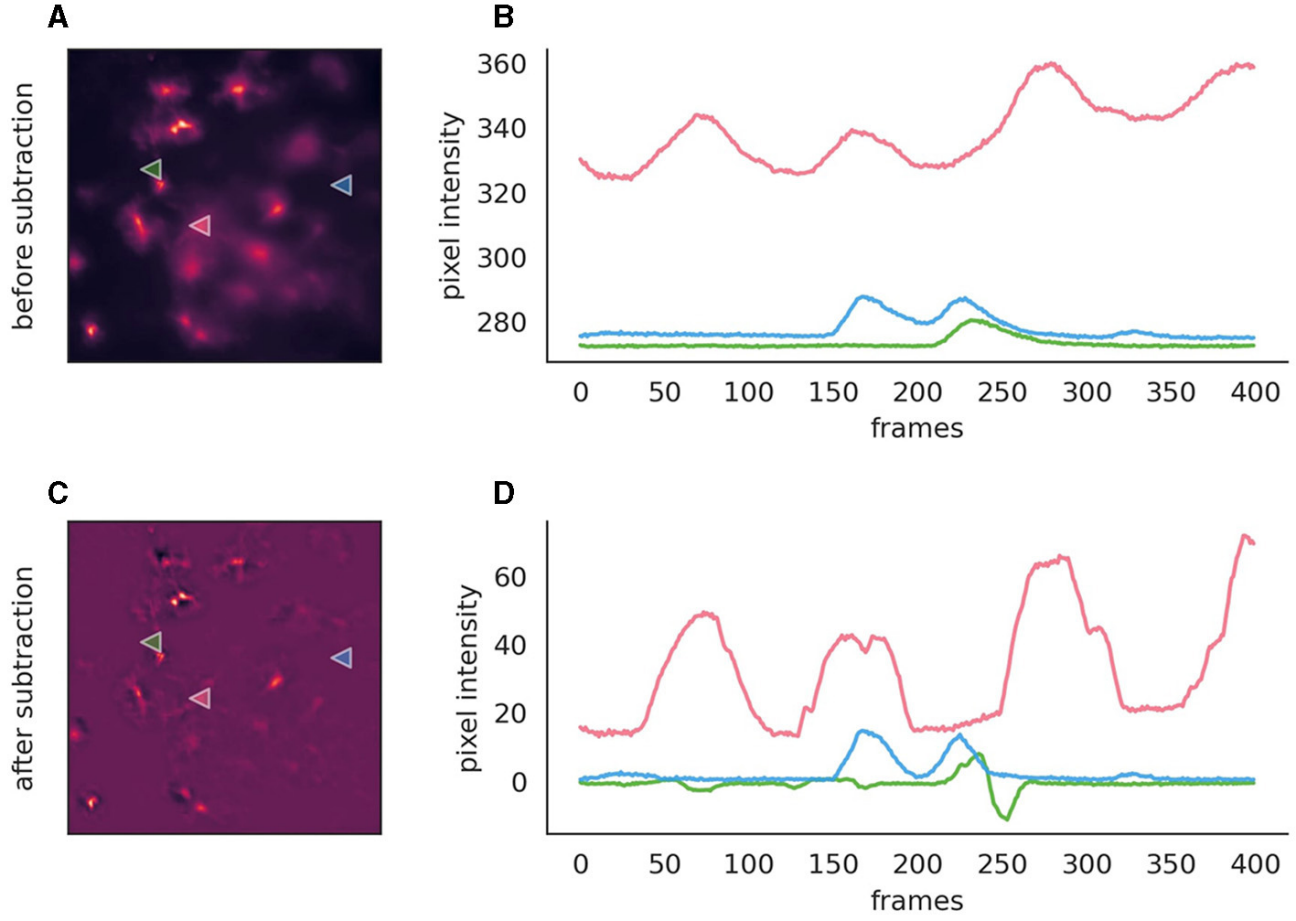
Comparison before and after the application of background subtraction to fluorescence imaging data. **(A, C)** Fluorescence images before and after background subtraction, respectively, illustrating the removal of extraneous noise. After subtraction, the background is close to zero and exhibits overall lower noise levels. **(B, D)** Intensity traces corresponding to the marked points on the images are plotted over time (400 frames, 50s). These traces highlight the efficacy of background subtraction, with the post-subtraction traces approaching zero on the y-axis, thereby indicating a substantial reduction of background, while signal amplitude is conserved. However, the signal shapes, especially in the red trace, exhibit slight alterations post-subtraction. Moreover, the green trace reveals the inadvertent introduction of spurious events, serving as a cautionary example of how background subtraction can inadvertently affect data integrity if parameters are not judiciously optimized.

Background subtraction, while optional, can significantly enhance event detection in fluorescence imaging by mitigating issues related to bright backgrounds, uneven lighting, or pronounced bleaching effects (Figure 4). AstroCAST leverages Radial Basis Function (RBF) interpolation for background approximation, ensuring accurate background modeling even in scenarios characterized by considerable signal fluctuations or missing data segments.

To facilitate optimal subtraction outcomes without introducing artifacts such as false positives or negatives, default parameters are provided, but fine-tuning may be necessary. An exploratory Jupyter notebook is available for guidance on how parameter adjustments influences the results.

The process begins with downsizing the video to manage computational load while preserving essential features. Peaks within each pixel's time series are identified and marked as NaN to exclude them from influencing the background model. The RBFInterpolator, implemented in SciPy (Virtanen et al., 2020), then estimates the background by interpolating these NaN values across the XYZ dimensions of the video. After resizing back to its original dimensions, the interpolated background is subtracted.

For initial image preparation, parameters such as –scale-factor for adjusting video resolution, –blur-sigma and –blur-radius for image blurring, are crucial. These adjustments are preparatory steps aimed at enhancing the effectiveness of the subsequent background subtraction process.

Peak detection is fine-tuned through parameters like –prominence, –wlen for window length, –distance between peaks, –width of peaks, and –rel-height defining the peak's cutoff height. These settings ensure precise identification of significant peaks, contributing to the accuracy of the background modeling.

The RBF interpolator constructs a smooth function from scattered data points by combining radial basis functions centered at data locations with a polynomial term (Fasshauer, 2007), adjusted by coefficients that solve linear equations to fit the data. Interpolation can be tunes with parameters such as –neighbors for the interpolation neighborhood, –rbf-smoothing for interpolation smoothness, –rbf-kernel to choose the kernel type, –rbf-epsilon, and –rbf-degree for kernel adjustment, facilitating a tailored approach to background modeling.

Background subtraction can be executed using either the Delta F (–method ‘dF') method, which directly subtracts the background, or the Delta F over F (–method ‘dFF') method, which divides the signal by the background post-subtraction. The choice between these methods depends on the specific requirements of the imaging data and the desired outcome of the subtraction process. However, it's important to consider that background subtraction may not be necessary for all datasets and could potentially introduce biases in event detection. Comparative analysis with and without this preprocessing step is recommended to assess its impact accurately (Supplementary material).


          > astrocast subtract-background --h5-loc ''mc/ch0''
          --method ''dF'' ''/path/to/file''

### 3.3 Event Detection

**Figure 5:**
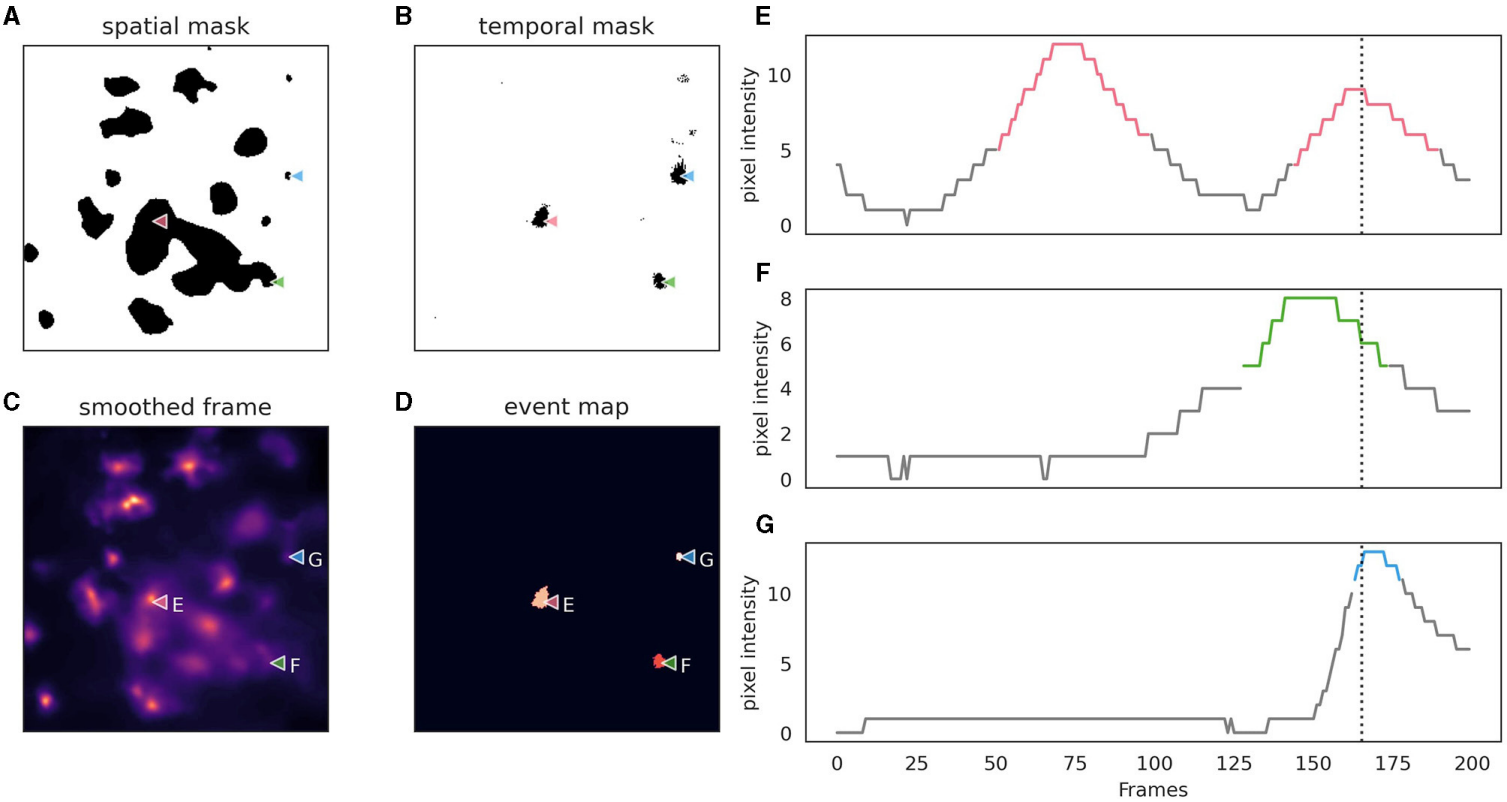
Depiction of astrocytic event detection employed by astroCaST using spatial and temporal thresholding. **(A)** Binary mask of frame after application of spatial threshold (min_ratio 1). **(B)** Binary mask of framer after application of temporal threshold (prominence 2, width 3, rel_height 0.9). **(C)** Frame used for thresholding after motion correction, denoising and smoothing. **(D)** Events detected as identified by both spatial and temporal thresholding. **(E–G)** Pixel intensity analysis for selected pixels [as indicated in **(A–D)**], with active frames color-coded in the plots. The frame shown in **(A–D)** is indicated as a vertical dotted line.

AstroCAST employs an event-centric approach to event detection, adapted from the AQuA package (Wang et al., 2018), and leverages the capabilities of scikit-image and dask for enhanced processing and analysis (van der Walt et al., 2014; Dask Development Team, 2016). Building on the AquA algorithm, astroCaST incorporates both spatial and temporal thresholding techniques to identify and extract events effectively (Figure 5). Spatial thresholding is executed based on either a single frame or a small volume, facilitating the identification of significant patterns or features in space. Concurrently, temporal thresholding is implemented by scrutinizing individual pixels for peaks across the time dimension, essentially tracking fluorescence changes over time.

Following the thresholding process, a binary mask is generated, which is used to label connected pixels, considering the full 3D volume of the video. This allows for the integration of different frames in the z-dimension to constitute a single event in a 3D context, ensuring a comprehensive analysis of the events captured in the video. Upon successful event detection, our protocol generates a folder with a .roi extension, which houses all files related to the detected events, offering an organized and accessible repository for the extracted data.

The event detection step is the most critical aspect of astroCaST and is sensitive to the choice of parameters. We have therefore implemented an interactive interface to explore the performance of each step during event detection given the relevant options. AstroCAST will prompt a link to the browser-based interface. We recommend to select a short range of frames in the video file to keep processing time during exploration short.


          > astrocast explorer -- input path ''/path/to/file''
          -- h5-loc ''/dataset/name''

Once users are satisfied with the chosen parameters, they can be exported to a YAML config file, that can be provided to the astrocast CLI.

#### 3.3.1 Detection module description

The detection module in AstroCAST applies a comprehensive approach to identify astrocytic events by smoothing, thresholding, and applying morphological operations on fluorescence imaging data. This section outlines the key steps and parameters used in the process.

##### 3.3.1.1 Smoothing

AstroCAST incorporates a Gaussian smoothing kernel to enhance event detection while preserving spatial features. Adjusting the --sigma and --radius parameters allows for the refinement of the smoothing effect, ensuring that events are emphasized without compromising the integrity of spatial characteristics. The smoothed data then serve as the basis for subsequent detection steps, although users can opt out of smoothing if desired.

##### 3.3.1.2 Identifying active pixels

Thresholding in AstroCAST is executed in two phases, spatial and temporal, to classify pixels as active with high precision. Spatial thresholding evaluates the entire frame to distinguish active pixels, automatically determining a cutoff value. It incorporates the mean fluorescence ratio of active to inactive pixels to mitigate the incorrect identification of noise as events. The --min-ratio parameter sets the minimum ratio threshold, and the --z-depth parameter allows for the consideration of multiple frames to improve threshold accuracy. Temporal thresholding analyzes the video as a series of 1D time series, identifying peaks with a prominence above a user-defined threshold. Parameters such as --prominence, --width, --rel-height, and --wlen fine-tune the detection of events over time, enhancing the separation of true events from noise. Combining spatial and temporal thresholding, users can choose to merge or differentiate the active pixels identified by each method, optimizing event detection based on specific dataset characteristics.

##### 3.3.1.3 Morphological operations

To address artifacts resulting from thresholding, such as holes (false negatives) and noise (false positives), AstroCAST employs morphological operations. Parameters like --area-threshold and --min-size adjust the maximum hole size to fill and the minimum size of active pixel clusters, respectively. These operations, which also consider connectivity and the --z-depth parameter, refine the detection outcome by smoothing gaps and removing minor artifacts, enhancing the clarity and segmentation of detected events.

##### 3.3.1.4 Additional options

Additionally, AstroCAST provides the option to exclude the video border from active pixel detection (--exclude-border) to mitigate motion correction artifacts. An experimental feature (--split-events) is available for separating incorrectly connected events, further improving the accuracy of event detection.

#### 3.3.2 Evaluating detection quality

AstroCAST offers a streamlined approach for assessing the quality of event detection through its command-line interface.


          > astrocast view-detection-results ''/path/to/roi''

Researchers can visually inspect the detection outcomes. During this evaluation phase, it is crucial to determine whether the detection process has successfully identified all expected events, thereby minimizing false negatives, and to assess the correctness of these events to ensure that noise has not been misclassified as true events, which would indicate false positives. If the detection results are not satisfactory, revising the event detection parameters or adjusting settings in the preceding background subtraction step (Section 3.2.4) may be necessary to achieve improved accuracy. For a dynamic visualization that illustrates the successful identification of events we included an example video (Supplementary material).

#### 3.3.3 Comparison of astroCaST and AQuA

**Figure 6:**
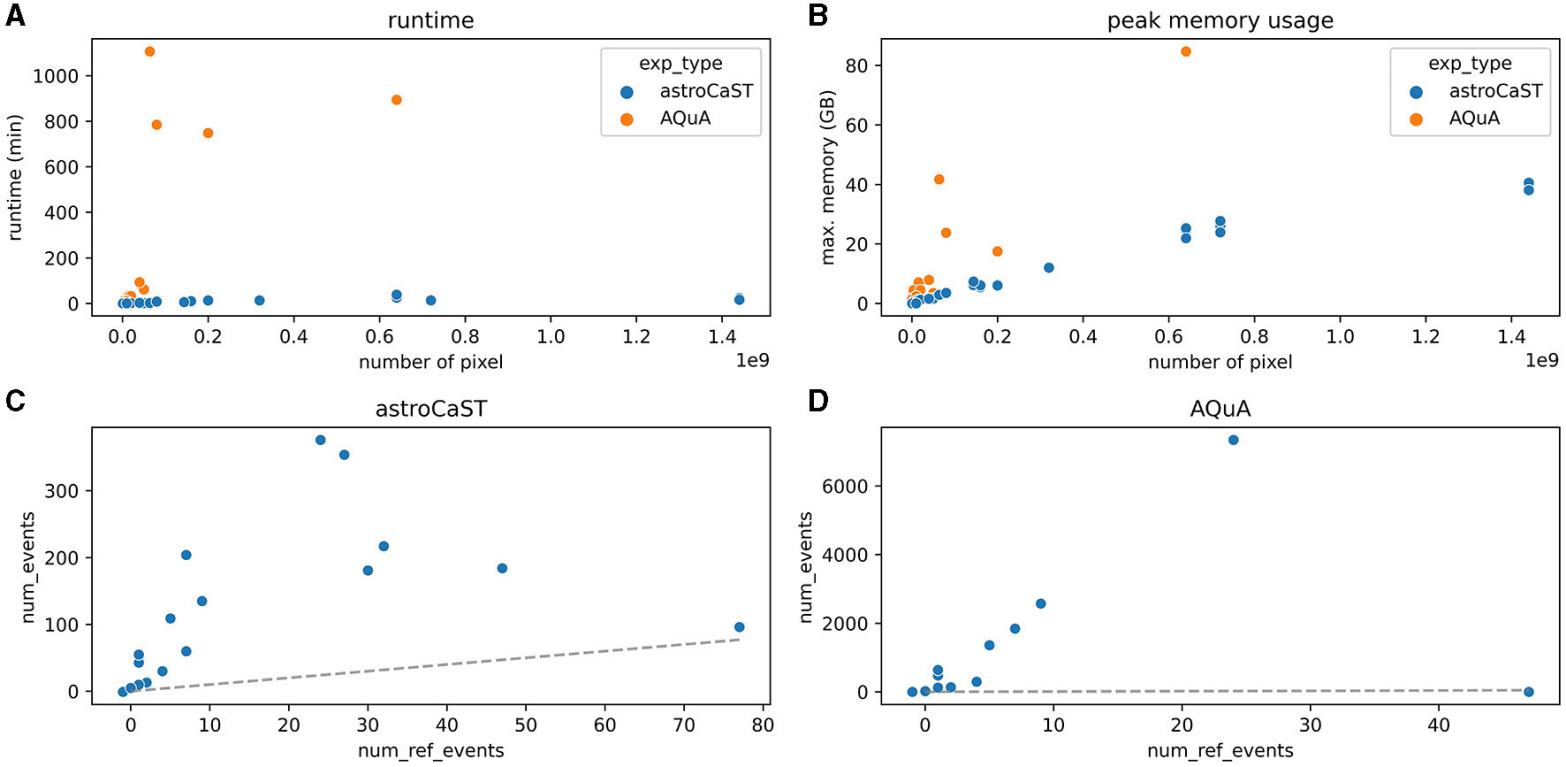
Performance comparison of astroCaST and AQuA on synthetic calcium imaging datasets. **(A, B)** Evaluation of computational efficiency for astroCaST and AQuA across synthetic video datasets of varying dimensions (100–5,000 frames, 100 × 100–1,200 × 1,200 pixels). **(A)** Graphical representation of runtime for each algorithm. **(B)** Analysis of peak memory consumption during the event detection phase. A run was considered failed if it exceeded available memory (76.8 GB). **(C, D)** Assessment of the accuracy in detecting astrocytic calcium events by astroCaST **(C)** and AQuA **(D)** without initial preprocessing steps. Detected events smaller than 5 and larger than 1,000 frames were excluded for visualization purposes. The dotted line marks the ideal correlation between the synthetic events generated and those identified by the algorithm.

We evaluated the performance of astroCaST against the current state-of-the-art astrocytic calcium imaging toolkit, AQuA, utilizing a tailored synthetic dataset. Our findings reveal that astroCaST was approximately ten times faster and using only half as much memory as AQuA (Figures 6A, B). A notable feature of astroCaST is its peak memory usage, which approaches a maximal limit, indicative of its efficient memory management facilitated by lazy data loading techniques. Both toolkits, however, exhibited tendencies to overestimate the number of detected events (Figures 6C, D). Despite this, the estimations made by astroCaST were consistently closer to the true values. These tests were conducted using default parameters for both toolkits to ensure an unbiased comparison. However, adjusting parameters tailored to the dataset can improve the detection accuracy of both toolkits. For example, filtering the detected events from astroCAST using the signal-to-noise ratio column yielded nearly identical detection matches to the generated events.

### 3.4 Embedding

#### 3.4.1 Quality control and standardization

After successful detection of events, users need to control the quality of the result to ensure data integrity for downstream analysis. In astroCaST this step optionally includes filtering and normalization. AstroCAST also features a dedicated GUI to guide selection of filter and normalization parameters.


          > astrocast exploratory-analysis --input-path
          ''/path/to/roi/'' --h5-loc ''/dataset/name''

Here, we are going to showcase the steps using the astroCaST python package directly. Users are directed to our example notebook to follow along. Of note, large datasets might require significant computational time. Hence, we recommend to utilize dynamic disk caching, via the cache_path parameter, to mitigate redundant processing.

        **from** pathlib **import** Path
        **from** astrocast.analysis **import** Events, Video
  
        # load video data
        video_path = 'path/to/processed/video.h5'
        h5_loc='df/ch0' lazy = False
        # flag to toggle on-demand loading;
        slower but less memory
        video = Video(data=video_path, loc=h5_loc,
        lazy=lazy)
  
        # load event data
        event_path = 'path/to/events.roi'
        cache_path=Path(event_path).joinpath('cache')
        eObj = Events(event_dir=event_path, data=video,
        cache_path=cache_path)

The events object has an events instance, which contains the extracted signal, as well as their associated attributes (for example ‘dz', ‘x0') and metrics (for example ‘v_area', ‘v_signal_to_noise_ratio'). An explanation of all columns can be found in the class docstring help(Events).

##### 3.4.1.1 Filtering

Typically, datasets undergo filtering to adhere to experimental parameters or to eliminate outliers. Although a detailed discourse on filtering protocols is outside this document's scope, we address several prevalent scenarios. For astrocytic events, imposing a cutoff for event duration is beneficial, especially when distinguishing between rapid and protracted calcium events. Moreover, applying a threshold for the signal-to-noise ratio helps to discard events with negligible amplitude. Although it seems optimal to include as many events as possible, practical issues such as computational limits and the risk of mixing important events with irrelevant data require a selective approach. Additionally, filtering aids in removing outliers, achieved either through manual adjustments or an unbiased clustering method. It is vital to recognize that the concept of an outlier is not universal, but intimately tied to the specifics of the experimental design. Rigorous, context-sensitive examination is crucial for accurate data interpretation. We encourage users to venture beyond the preset analysis tools of AstroCAST, engaging with their data through custom analysis methods tailored to their unique experimental conditions.

          **from** astrocast.analysis **import** Plotting
          plot = Plotting(events=eObj)
  
*          # explore distribution*
          plot.plot_distribution(column='dz',
          outlier_deviation=3)
  
*          # filter based on user choice*
*          #  here: events of length between 3 and 200 frames*
          filter_choice = {'dz': (3, 200)}
          eObj.filter(filter_choice, inplace=True)

##### 3.4.1.2 Event extension

AstroCAST's event detection module is designed to pinpoint individual events with precise temporal accuracy. Often, this precision means that the peripheral regions, or shoulders, of the event peak might not be captured. Yet, these initial and concluding slopes of the event can reveal crucial information about its nature. To address this, astroCaST includes a feature that allows for the extension of detected events to include adjacent frames.


         # extend events
         # use_footprint: toggles between maximum projection
         (footprint) **or** first/last event frame
         # extend: either an int, (int, int) or -1 for
         extension across the full video
         eObj.get_extended_events(use_footprint=True,
         extend=3, in_place=True, load_to_memory=True)

##### 3.4.1.3 Enforcing fixed-length events

Enforcing a fixed event length is a requirement for some modules of astroCaST. However, enforcing a length will significantly distort the actual event characteristics. Use length enforcement cautiously, as it can alter your data and even overlap with other nearby events. If required by your analysis, the toolkit will prompt you to unify event lengths (for example CNN Autoencoders). For diverse event durations, consider filtering your data for similar lengths or analyzing short and long events separately to maintain data integrity.

*          # extend events*
          eObj.get_extended_events(enforce length=15,
                      use_footprint=True, extend=8,
                      in_place=True, load_to_memory=True)

##### 3.4.1.4 Normalization and standardization

The final step before clustering involves normalizing the signal. This process aims to mitigate the impact of technical effects encountered during recordings, such as fluorescence bleaching, and facilitating the comparison across multiple experiments. Normalization is particularly critical when employing machine learning algorithms, as it ensures data uniformity and comparability.

AstroCAST offers a versatile normalization interface to accommodate various research needs. This dynamic system allows users to apply a series of operations such as subtraction, division, and first-order differentiation to standardize data. Operations can be tailored using common statistical functions like the mean, median, or standard deviation. Additionally, AstroCAST provides a ‘population_wide' flag, offering users the choice to normalize signals either individually or across a collective dataset, thereby maximizing analytical precision and flexibility.

*       # define normalization instructions*
*       #  here: take the differential of each signal*
*       #  and then divide by maximum value in the population*
       instructions = {
           0: [''diff'', {}],
           1: [''divide'', {''mode'': ''max'',
           ''population_wide'': True}]
       }
  
*          # apply the normalization*
          eObj.normalize(instructions, inplace=True)

#### 3.4.2 Event encoding

AstroCaST introduces a structured approach to address the challenge of varying event durations, detailed here with increasing complexity. The normalized timeseries are transformed into fixed-length feature vectors suitable for clustering and further analysis. This sequence of steps allows for a more focused analysis while balancing the need for data integrity and computational efficiency. Each alternative has advantages and disadvantages, notably the ability to deal with variable length events (Table 4). It is essential to re-train the encoding model whenever the dataset is changed, to ensure optimal encoding.

**Table 4:** Evaluation of different methodologies for embedding and distance calculations.

	**Feature**	**Convolutional**	**Recurrent**	**Pearson**	**Dynamic time**
	**Extraction**	**Autoencoder**	**Autoencoder**	**Correlation**	**Warping**
Abbreviation	**FExt**	**CNN**	**RNN**	**pearson**	**DTW**
Fixed length	—	✓	—	—	—
Normalization	—	✓	✓	—	—
Training	—	✓	✓	—	—
Memory limited	—	—	—	✓	✓
Wall time limited	✓	—	—	✓	✓
Parallelized	—	—	—	✓	◐

Highlights the requirements and limitations. A (✓) indicates a necessary requirement, items with (—) are optional and a (◐) indicates a difference between operating systems.

##### 3.4.2.1 Feature extraction

Feature extraction offers a simple yet powerful approach to reduce timeseries data into a manageable set of dimensions. The module employs a variety of metrics, like mean, median, and Shannon entropy, accessible through help(FeatureExtraction). While this method is length-agnostic, it may overlook key features, due to the inherent dimensional reduction. Further dimension reduction can be applied using techniques like PCA, t-SNE, or UMAP.

          **import** astrocast.reduction as red
  
*          # extract features*
          fe = red.FeatureExtraction(eObj)
          features = fe.all_features(dropna=True)
  
*          # classify labels*
          hdb = clust.HdbScan()
          lbls = hdb.fit(features)
  
*          # visualize with UMAP*
          umap = red.UMAP()
          embedding = umap.train(features)
          umap.plot(data=embedding, labels=lbls,
          use_napari=False)

##### 3.4.2.2 CNN Autoencoder

Using a CNN Autoencoder provides an advanced way to maintain key features while reducing data complexity. The CNN Autoencoder consists of an encoder that maps the timeseries to a latent vector and a separately trained decoder, which performs the inverse operation. After training, only the encoder is utilized for event embedding. Note that this approach requires the input to have a fixed length (see Section 3.4.1.3).

          **import** astrocast.autoencoders as AE
  
*          # check that traces have fixed length*
          if eObj._is_ragged():
              **raise** ValueError()
          target_length = len(eObj.events.iloc[0].trace)
  
*          # Prepare data*
          data = np.array(eObj.events.trace.tolist())
          X_train, X_val, X_test = cnn.split_dataset
          (data=data)
  
*          # create and train model*
          cnn = AE.CNN_Autoencoder(target_length=target_
          length)
         cnn.train_autoencoder(X_train=X_train, X_val=X_val,
         epochs=25)
  
*         # save model*
         cnn.save(''cnn.pth'')
  
*         # evaluate embedding performance*
         cnn.plot_examples_pytorch(X_test, show_diff=True)

##### 3.4.2.3 Recurring Neural Network (RNN) Autoencoder

For encoding time series of variable lengths into consistent latent vectors, the RNN Autoencoder stands out. This approach is notably advantageous over CNN embeddings, as it eliminates the need for uniform input lengths, offering a more flexible and comprehensive encoding solution for diverse data sets. However, this flexibility comes at the cost of increased computational demand, complexity in parameter tuning and an overall decrease in embedding quality.

          **import** astrocast.autoencoders as AE
  
*          # Prepare data*
          pdl = AE.PaddedDataLoader(data=eObj.events.trace)
          X_train, X_val, X_test = pdl.get_datasets(batch_
          size=16, val_size=0.1, test_size=0.05)
          model_path = ''path/to/save/models/''
  
*          # train RNN*
          tRAE = AE.TimeSeriesRnnAE(use_cuda=True)
          tRAE.train_epochs(dataloader_train=X_train,
                            dataloader_val=X_val,
                            num_epochs=10,
                  *          # number of training epochs*
                            patience=10,
                  *          # epochs to wait before early*
                            stopping safe_after_epoch=
                            model_path
                            )
  
*          # save model*
          tRAE.save_models(''encoder.pth'', ''decoder.pth'')
  
*          # evaluate embedding performance*
          fig, x_val, y_val, latent, losses = tRAE.plot_
          traces(dataloader=X_test, figsize=(20, 20))
          fig.savefig(''tRAE_performance.png'')

### 3.5 The outcomes of experiments

The last step of the protocol is to evaluate the outcomes of experiment (Table 5), utilizing various packages including scikit-learn and dtaidistance (Pedregosa et al., 2011; McInnes et al., 2017, 2018; Meert et al., 2020). This section provides examples to inspire various ways datasets can be analyzed. The best analysis approach will depend on the nature of the study, thus we include examples of common types of experiments (Figure 6). AstroCAST is designed to be flexible and modular, allowing for custom addition of analyses tailored to the specific dataset. The examples in this section focus on two primary types of analyses: comparing groups under different conditions (Conditional Contrasts) and detecting coincidences of independent stimuli (Coincidence Detection). When analyzing data from multiple experiments (for example, different conditions) the datasets can be combined with the MultiEvents class.

**Table 5:** Comprehensive overview of experiment types that are included in astroCaST, with their respective requirements.

	**Conditional contrasts**		**Coincidence detection**	
	**Classifier**	**Hierarchical**	**Classifier**	**Regression**
Training required	✓	—	✓	✓
User labels	Group	Group	Timing	Timing
Input type	Embedding	Distance	Embedding	Embedding

        **from** astrocast.analysis **import** MultiEvents
  
*        # collect list of experiments*
        paths = [''./path/1'', ''./path/2'', ...]
  
*        # optional: define group membership*
        groups = [''group_1'', ''group_2'']
  
*        # load combined dataset*
        combined_events_obj = MultiEvents(events_dirs=paths,
        groups=groups,
                                           data=''infer'',
                                           lazy=True)

Here, we are using sets of synthetic datasets to reproducibly show the advantages and disadvantages of each analysis approach. We encourage users to explore their own variations of synthetic datasets.

          **from** astrocast.helper **import** DummyGenerator,
          SignalGenerator
  
*          # general settings*
          z_range = (0, 10000)
*          # range of frames that events can be created*
  
*          # Create signal generators*
          group_1 = SignalGenerator(trace_length=(50, 50),
                      noise_amplitude=0.001,
                      parameter_fluctuations=0.01,
                      b=1, plateau_duration=1)
          group_2 = SignalGenerator(trace_length=(50, 50),
                      noise_amplitude=0.001,
                      parameter_fluctuations=0.01,
                      b=2, plateau_duration=6)
          generators = [group_1, group_2]
  
*          # Create stimulus timings*
          timing_1 = None  *# random event distribution*
          timing_2 = list(range(0, z_range[1], 1000))
          timings = [timing_1, timing_2]
  
*          # Create synthetic events*
          dg = DummyGenerator(name=''synthetic_events'',
              num_rows=100  # *number of events per group*
              z_range=z_range, generators=generators,
              timings=timings,
          )
          eObj = dg.get_events()
  
*          # create embedding as in previous steps*

#### 3.5.1 Conditional contrasts

**Figure 7:**
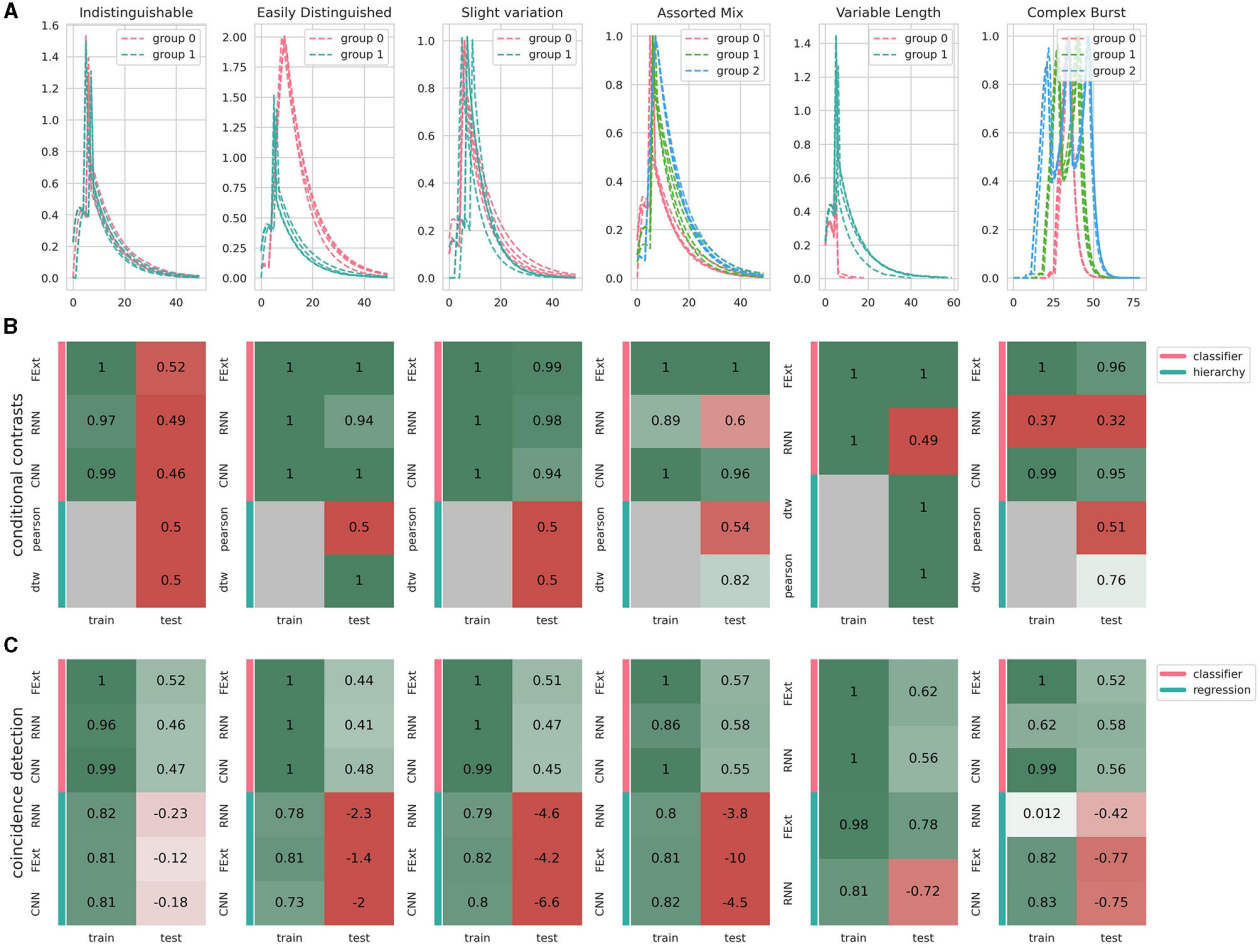
Performance of different algorithms on analyzing various synthetic satasets. **(A)** Showcase of synthetic calcium events designed to represent various levels of analytical difficulty, where color coding corresponds to events generated under different parameter sets that simulate diverse conditions or event types. All events include a random noise amplitude of 0.001 and parameter fluctuations of 0.01, subtly varying each event's parameters. **(B)** Conditional contrasts analysis assesses algorithmic efficiency in distinguishing events from differing conditions (groups 0, 1, and 2). Events are characterized using different methods: FExt for Feature Extraction, CNN for Convolutional Autoencoder, and RNN for Recurrent Autoencoder. The hierarchical clustering leverages distance metrics between events (Pearson correlation or dynamic time warping), depicted by the absence of training dependency in gray. CNN's inability to process variable-length events results in its omission in the final panel. **(C)** Coincidence detection analysis gauges algorithm performance in predicting the occurrence of stimulus events. This encompasses two groups: one with events exclusively occurring during a stimulus and another with randomly occurring events. The embedding classifier and prediction methods are consistent with **(B)**, where the classifier identifies stimulus occurrence, and regression determines the exact timing of the stimulus in coinciding events. Displayed scores represent the lowest achieved in three replicates, adhering to cross-validation principles.

This module compares different experimental groups by assessing whether the observed effects are condition-dependent (Figure 7B). Common examples include application of different drugs, samples from different animal models or different brain regions.

##### 3.5.1.1 Classifier (predict the condition)

Use classification algorithms to predict the condition of each event based on its features. This provides an indication of how effectively the conditions can be distinguished based on the observed events. Feature embedding is necessary.

          **from** astrocast.clustering **import** Discriminator
  
*         # assumes eObj and embedding was created previously*
         categories = eObj.events.group.tolist()
         classifier = ''RandomForestClassifier''
  
*         # create disciminator object and train*
         discr = Discriminator(eObj)
         clf = discr.train_classifier(embedding=embedding,
                                      category_vector=
                                      categories,
                                      classifier=classifier)
  
*         # evaluate the outcome (metrics and confusion*
         matrix)
         scores = discr.evaluate(show_plot=False)
         **print**(scores)

##### 3.5.1.2 Hierarchical clustering (overlap with conditions)

Hierarchical clustering groups similar events together. Similarity between event traces is assessed by either Dynamic Time Warping (DTW) or Pearson Correlation, basically calculating a distance between all events. The aim is to see if these groups correspond to different experimental conditions, which would indicate that the conditions have distinct profiles. Hierarchical clustering does not depend on feature embedding and can be safely performed on the normalized event traces. The Linkage module will return barycenters, which can be understood as a consensus event shape for each cluster, and a cluster_lookup_table which maps the identified clusters to the events. Clusters can be defined by either a set number of expected clusters (criterion=‘maxlust', cutoff=expected_num_clusters) or maximum distance (criterion=‘distance',cutoff=max_distance).

          **from** astrocast.clustering **import** Linkage,
          Discriminator
  
*          # settings*
          categories = eObj.events.group.tolist()
          correlation_type = ''dtw''  *# or ‘pearson'*
  
*         # create linkage object and create clusters*
         link = Linkage()
         result = link.get_barycenters(eObj.events,
           cutoff=num_groups,
           criterion='maxclust',
           distance_type
           =correlation_type
           )
*         # evaluate outcome (metrics and confusion matrix)*
         barycenters, cluster_lookup_table = result
         eObj.add_clustering(cluster_lookup_table,
         column_name='predicted_group')
         predicted_categories = eObj.events.predicted_group.
         tolist()
  
         scores = Discriminator.compute_scores(categories,
         predicted_categories,
           scoring=
           ''clustering'')
         **print**(scores)

#### 3.5.2 Coincidence detection

This module focuses on identifying whether certain conditions or events coincide with or predict other phenomena (Figure 7C). Common examples would be neuronal bursts or observed animal behavior.

##### 3.5.2.1 Classifier (predict incidence occurred)

Similar to the classifier used in conditional contrasts, this module predicts whether a specific incidence or event has occurred. This is particularly useful for identifying causal relationships or triggering events.

          **from** astrocast.clustering **import**
          CoincidenceDetection
  
*          # assumes eObj and embedding was created and the*
          stimulus timings are known
          cDetect = CoincidenceDetection(events=eObj,
                                        incidences=timing,
                                        embedding=embedding)
  
*          # predict classes and evaluate outcome*
          _, scores = cDetect.predict_coincidence
          (binary_classification=True)
          **print**(scores)

##### 3.5.2.2 Regression (timing of incidence; coinciding events only)

Regression analysis can be used to predict when an incidence occurred based on the event embedding. This analysis aims to establish whether astrocytic events correlate with the incident in question, or vice versa.

          **from** astrocast.clustering **import**
          CoincidenceDetection
  
*          # assumes eObj and embedding was created and the*
          stimulus timings are known
          cDetect = CoincidenceDetection(events=eObj,
                                        incidences=timing,
                                        embedding=embedding)
  
*          # predict classes and evaluate outcome*
          _, scores = cDetect.predict_incidence_location()
          print(scores)

## 4 Discussion

In the field of neurobiology, it is important to acknowledge the substantial gap in open-source, customizable tools tailored for the comprehensive characterization and dynamics of astrocytes. This scarcity significantly hinders the advancement of our understanding of the complex functions carried out by these glial cells. Their function extends far beyond their traditional role of supporting neuronal activity (Ransom et al., 2003; Montalant et al., 2021). Thus, the development and refinement of specialized tools such as astroCaST represents a step forward in addressing this limitation. Unlike previous methodologies that regard astrocytes as mere adjuncts to neuronal studies, astroCaST aimed to meticulously and explicitly address the uniqueness of astrocyte research (Table 1).

AstroCAST offers comprehensive options for analyzing and interpreting activity events repurposing existing tools from various data science domains for astrocyte research. This approach highlights the importance of leveraging accumulated knowledge across fields to enhance the efficiency and breadth of astrocyte studies. Moreover, the advances in bioimaging dataset processing are still lagging behind other big data analysis approaches. Addressing this gap, astroCaST is a dedicated machine-learning driven toolkit that can be used to identify, denoise and characterize the dynamics of a set cell population. Compared to other available tools, astroCaST was specifically developed for astrocytes [Suite2P (Pachitariu et al., 2017), CaImAn (Giovannucci et al., 2019), and CaSCaDe (Rupprecht et al., 2021)] and is open-source [CHIPS (Barrett et al., 2018), AQuA (Wang et al., 2018), Begonia (Bjørnstad et al., 2021), and MSparkles (Stopper et al., 2022)]. Moreover, it includes timeseries clustering which none of these tools provide. Specifically, astroCaST is tailored for astrocytes by being agnostic to the spatial location of events. It first identifies active pixels across the dataset and subsequently groups these pixels into coherent events, a methodological approach similar to that used by AQuA (Wang et al., 2018), thereby enhancing its applicability and accuracy in astrocytic calcium imaging studies.

We conducted a comparative analysis of event detection capabilities between astroCaST and AQuA, two advanced toolkits for astrocytic calcium imaging (Figure 6). Notably, AQuA encountered limitations related to both memory and computational time. In contrast, astroCaST demonstrated a robust performance, thereby allowing for the analysis of significantly larger datasets. Both astroCaST and AQuA tended to overestimate the number of detected events. This characteristic is advantageous when using default parameters as we did, as it is preferable to detect more events, which can subsequently be filtered, than missing potential events. Missing events would necessitate re-running the analysis, increasing both the computational burden and the time to results. While our comparison utilized a synthetic dataset, which is advantageous for controlled testing and benchmarking, this highlights the need for publicly available, expert-scored experimental data. Access to such datasets would facilitate more robust tool development and validation, ensuring that these computational tools can be reliably used across various research settings. The comparative analysis reveals that both toolkits excel with small datasets, yet astroCAST offers significant advantages in speed, memory efficiency, and accuracy when handling larger datasets.

We also assessed how different embeddings and analysis approaches handle the intricate dynamics of astrocytic calcium events. To address method performance we focused on accuracy, generalization to new data, and computational efficiency (Figure 6). In evaluating the robustness of the event encoding methods, we generated three synthetic datasets, performed the analysis, and chose the lowest score for each method and embedding variant. This approach provides a lower boundary of performance, demonstrating the robustness of our methods to varying datasets and conditions. We first tested two control groups of signals derived from the same signal population. This design ensures that both groups are indistinguishable, as they possess identical characteristics. This dataset validates that astroCaST does not access or infer information beyond what is present in the data itself. Therefore, while astroCaST can memorize details from the training dataset, it appropriately fails to generalize this memorization to unseen, yet identical, data.

Feature extraction (FExt) emerges as a quick and straightforward method, albeit less effective with complex event types. Convolutional Neural Networks (CNNs) offer speed and accuracy but falter with astrocytic events' intrinsic variable lengths. Attempts to enforce fixed lengths or extend events risk altering their inherent shapes and producing spurious results. Recurrent Neural Networks (RNNs) show promise in dealing with variable event lengths but are challenging to fine-tune. This can be seen by their subpar performance in the triplet condition (accuracy of 0.56). Pearson correlation analysis proved inadequate, with all clustering algorithms failing to differentiate between events with significant variances (0.5 prediction accuracy), except for the trivial case involving variable lengths. Dynamic Time Warping (DTW) excels in handling variable lengths but is slower and slightly less accurate (0.94 accuracy compared to others' 0.99) for fixed-length events. However, avoiding an embedding step compensates for the slower processing speed, albeit with a significant memory constraint as the number of events increases [O(*n*^2^) complexity]. In summary, while each approach has its strengths and weaknesses in processing astrocytic calcium imaging datasets, our findings highlight the crucial balance between accuracy, adaptability to variable event lengths, and computational demands.

The intended audience of astroCaST are bioinformaticians or neuroscientists with a solid foundation in programming. Proficiency in Python and familiarity with executing command line tools are essential prerequisites for effectively utilizing this toolkit. Importantly, troubleshooting code is a necessary skill set for circumnavigating potential challenges during data processing and personalizing experimental implementation. Prior experience in image analysis, time series clustering, and machine learning is advantageous but not mandatory. However, to decode the intricacies of astrocytes, it is essential employ a multidisciplinary team skilled in both experimental neuroscience and computational analysis.

In our experience, astroCaST's GUI responsiveness was a key limitation, indicating an area of fine-tuning for future versions. Furthermore, the toolkit could not pass the threshold of “inference-of-cell” implementation, which would assign events to individual cells. Thus, the “Functional Units” module currently exists as an experimental feature, but it lacks proper validation and testing due to insufficient biological data (e.g., sparse GCaMP6f expression recordings or post-imaging staining).

However, astroCaST can facilitate several key outcomes for researchers working with astrocytic calcium imaging data analysis. On the technical front, the tool is designed to handle large-scale datasets effectively. For example, in time-lapse recordings of astrocytes at 20X magnification and 1,024 × 1,024 pixel resolution, astroCaST is equipped to identify up to 1,000 astrocytic events per 100 frames. As the analysis progresses, users should find that the software can comfortably manage up to 100,000 events using the algorithms provided. This performance equates to the comparative analysis of around 7 time-lapse videos, each with a 20-minute recording time. Future work will highlight the biological insights and capabilities of astroCAST.

The astroCaST toolkit has proved to be effective in analyzing calcium events in fluorescence imaging data. We have tested the system in depth on acute slices, as well as available public data for *in-vivo* and cell culture recordings (Table 3). There are, however, challenges that might be considered as limitations. For example, due to its design agnosticism toward cell shape, astroCaST cannot firmly attribute events to individual cell. This allows for the analysis of recordings even when cells overlap or change morphology. Furthermore, issues that originate from the difficulty to distinguish close bordering events, the computational complexity, the event length and data dimensionality, and others, may also need fine-tuning. We acknowledge that users might encounter some of these, and we make suggestions for each in the troubleshooting section. We regard astroCaST's flexible modular design as crucial for overcoming challenges, empowering researchers to translate astrocytic calcium signaling into biological insights.

AstroCAST is designed to handle larger datasets, analyzing more events than previously possible in astrocyte-specific studies with enhanced efficiency. This is achieved without compromising computational time. From an innovation standpoint, astroCaST offers more than just event detection. Users can expect a cohesive, end-to-end analytical workflow that guides them from raw video data to insightful experimental conclusions. The software brings structure and flexibility to the often-challenging variable-length timeseries analysis, setting it apart as a unique tool in the field of astrocytic calcium imaging. AstroCAST not only enhances our ability to probe astrocyte activity, but also holds the promise of unveiling previously unexplored aspects of their role in neural circuitry and brain development and function. This demonstrates the potential for future synergistic integration of advanced imaging techniques, such as calcium imaging, with established and high-resolution methods like immunohistochemistry or spatial transcriptomics, offering a wealth of new possibilities for in-depth astrocytic analysis.

## Use of Generative AI

During the preparation of this manuscript, we utilized the generative capabilities of OpenAI's ChatGPT-4. The AI's primary role was to assist in proofreading and language refinement to ensure clarity, conciseness, and readability of the text. While the AI provided suggestions for language adjustments, the final manuscript content was thoroughly reviewed, curated, and approved by the authors. We, the authors, take full and complete responsibility for the integrity and accuracy of the manuscript content.

## Data availability statement

The raw data supporting the conclusions of this article will be made available by the authors, without undue reservation.

## Ethics statement

The animal study was approved by Stockholm Animal Research Ethics Committee (approval numbers 15819-2017, 19026-2022). The study was conducted in accordance with the local legislation and institutional requirements.

## Author contributions

JR: Data curation, Formal analysis, Methodology, Project administration, Software, Validation, Visualization, Writing – original draft, Writing – review & editing, Conceptualization. AG-S: Software, Writing – original draft, Writing – review & editing. AS: Project administration, Supervision, Writing – original draft, Writing – review & editing. EH: Project administration, Funding acquisition, Supervision, Writing – original draft, Writing – review & editing, Conceptualization.
