# Supplementary material for: Astrocytic Calcium Signaling Toolkit (astroCaST): efficient analysis of dynamic astrocytic calcium events
Source: Front Cell Neurosci. 2024 Jun 10;18:1408607. doi: 10.3389/fncel.2024.1408607 (PMC11195029; doi:10.3389/fncel.2024.1408607)
Supplement: Supplementary file 3 [file Data_Sheet_1.PDF]

Supplementary Material

0.1 Figures

Figure S1. Comparison of Imaging Data Before and After Background Subtraction in the pre-Bötzinger Complex (preBötC). This figure juxtaposes the original imaging data (left) with the data following background subtraction (right), captured at 8Hz, to demonstrate the enhancement in clarity and contrast after subtraction.

Figure S2. Detection of Astrocytic Events over Time in the pre-Bötzinger Complex (preBötC). This video showcases the detection of astrocytic events within the preBötC, captured at 8Hz over one minute of recording time. Visualized through contours, each detected event is highlighted, with different colors marking separate events to clearly delineate the spatial and temporal unfolding of astrocytic activity.
